# Supplementary material for: Pyruvate Dehydrogenase Kinase 1 inhibition mediated oxidative phosphorylation enhancement in cartilage promotes osteoarthritis progression
Source: BMC Musculoskelet Disord. 2023 Jul 20;24:597. doi: 10.1186/s12891-023-06585-6 (PMC10357736; doi:10.1186/s12891-023-06585-6)
Supplement: Supplementary file 1 — Additional file 1. [file 12891_2023_6585_MOESM1_ESM.zip › KEGG.Gsea.1653622432464/KEGG_PRION_DISEASES.html]

Details for gene set KEGG\_PRION\_DISEASES[GSEA]

|  || Dataset | 55457\_collapsed\_to\_symbols.cls.cls #OA\_versus\_normal.cls.cls #OA\_versus\_normal\_repos |
| Phenotype | cls.cls#OA\_versus\_normal\_repos |
| Upregulated in class | normal |
| GeneSet | KEGG\_PRION\_DISEASES |
| Enrichment Score (ES) | -0.30668205 |
| Normalized Enrichment Score (NES) | -1.0682154 |
| Nominal p-value | 0.37392795 |
| FDR q-value | 0.5686505 |
| FWER p-Value | 1.0 |
Table: GSEA Results Summary

  

Fig 1: Enrichment plot: KEGG\_PRION\_DISEASES      
 Profile of the Running ES Score & Positions of GeneSet Members on the Rank Ordered List

  

| SYMBOL | TITLE | RANK IN GENE LIST | RANK METRIC SCORE | RUNNING ES | CORE ENRICHMENT || 1 | MAPK3 | mitogen-activated protein kinase 3 [Source:HGNC Symbol;Acc:HGNC:6877] | 757 | 0.579 | -0.0052 | No |
| 2 | PRKACB | protein kinase cAMP-activated catalytic subunit beta [Source:HGNC Symbol;Acc:HGNC:9381] | 771 | 0.576 | 0.0471 | No |
| 3 | C8B | complement C8 beta chain [Source:HGNC Symbol;Acc:HGNC:1353] | 1001 | 0.517 | 0.0772 | No |
| 4 | NCAM2 | neural cell adhesion molecule 2 [Source:HGNC Symbol;Acc:HGNC:7657] | 2085 | 0.352 | 0.0256 | No |
| 5 | SOD1 | superoxide dismutase 1 [Source:HGNC Symbol;Acc:HGNC:11179] | 2413 | 0.313 | 0.0292 | No |
| 6 | C1QA | complement C1q A chain [Source:HGNC Symbol;Acc:HGNC:1241] | 2902 | 0.260 | 0.0154 | No |
| 7 | ELK1 | ETS transcription factor ELK1 [Source:HGNC Symbol;Acc:HGNC:3321] | 3357 | 0.216 | 0.0001 | No |
| 8 | C1QB | complement C1q B chain [Source:HGNC Symbol;Acc:HGNC:1242] | 3365 | 0.215 | 0.0195 | No |
| 9 | C8A | complement C8 alpha chain [Source:HGNC Symbol;Acc:HGNC:1352] | 3918 | 0.163 | -0.0083 | No |
| 10 | C7 | complement C7 [Source:HGNC Symbol;Acc:HGNC:1346] | 4078 | 0.148 | -0.0069 | No |
| 11 | C5 | complement C5 [Source:HGNC Symbol;Acc:HGNC:1331] | 4387 | 0.122 | -0.0196 | No |
| 12 | C9 | complement C9 [Source:HGNC Symbol;Acc:HGNC:1358] | 5415 | 0.039 | -0.0957 | No |
| 13 | CCL5 | C-C motif chemokine ligand 5 [Source:HGNC Symbol;Acc:HGNC:10632] | 6584 | -0.048 | -0.1820 | No |
| 14 | MAPK1 | mitogen-activated protein kinase 1 [Source:HGNC Symbol;Acc:HGNC:6871] | 6853 | -0.068 | -0.1966 | No |
| 15 | NCAM1 | neural cell adhesion molecule 1 [Source:HGNC Symbol;Acc:HGNC:7656] | 7012 | -0.081 | -0.2014 | No |
| 16 | C6 | complement C6 [Source:HGNC Symbol;Acc:HGNC:1339] | 7316 | -0.103 | -0.2154 | No |
| 17 | IL1A | interleukin 1 alpha [Source:HGNC Symbol;Acc:HGNC:5991] | 7842 | -0.144 | -0.2429 | No |
| 18 | HSPA1A | heat shock protein family A (Hsp70) member 1A [Source:HGNC Symbol;Acc:HGNC:5232] | 7876 | -0.146 | -0.2319 | No |
| 19 | STIP1 | stress induced phosphoprotein 1 [Source:HGNC Symbol;Acc:HGNC:11387] | 8559 | -0.198 | -0.2665 | No |
| 20 | C8G | complement C8 gamma chain [Source:HGNC Symbol;Acc:HGNC:1354] | 8871 | -0.223 | -0.2701 | No |
| 21 | PRNP | prion protein [Source:HGNC Symbol;Acc:HGNC:9449] | 9042 | -0.235 | -0.2615 | No |
| 22 | BAX | "BCL2 associated X, apoptosis regulator [Source:HGNC Symbol;Acc:HGNC:959]" | 9323 | -0.257 | -0.2594 | No |
| 23 | MAP2K2 | mitogen-activated protein kinase kinase 2 [Source:HGNC Symbol;Acc:HGNC:6842] | 9932 | -0.315 | -0.2775 | Yes |
| 24 | FYN | "FYN proto-oncogene, Src family tyrosine kinase [Source:HGNC Symbol;Acc:HGNC:4037]" | 10092 | -0.330 | -0.2593 | Yes |
| 25 | MAP2K1 | mitogen-activated protein kinase kinase 1 [Source:HGNC Symbol;Acc:HGNC:6840] | 10197 | -0.340 | -0.2358 | Yes |
| 26 | PRKACG | protein kinase cAMP-activated catalytic subunit gamma [Source:HGNC Symbol;Acc:HGNC:9382] | 10466 | -0.370 | -0.2224 | Yes |
| 27 | PRKACA | protein kinase cAMP-activated catalytic subunit alpha [Source:HGNC Symbol;Acc:HGNC:9380] | 10611 | -0.384 | -0.1980 | Yes |
| 28 | LAMC1 | laminin subunit gamma 1 [Source:HGNC Symbol;Acc:HGNC:6492] | 10642 | -0.387 | -0.1645 | Yes |
| 29 | HSPA5 | heat shock protein family A (Hsp70) member 5 [Source:HGNC Symbol;Acc:HGNC:5238] | 10820 | -0.407 | -0.1405 | Yes |
| 30 | PRKX | protein kinase X-linked [Source:HGNC Symbol;Acc:HGNC:9441] | 11634 | -0.522 | -0.1553 | Yes |
| 31 | EGR1 | early growth response 1 [Source:HGNC Symbol;Acc:HGNC:3238] | 12052 | -0.607 | -0.1315 | Yes |
| 32 | IL1B | interleukin 1 beta [Source:HGNC Symbol;Acc:HGNC:5992] | 12105 | -0.622 | -0.0779 | Yes |
| 33 | IL6 | interleukin 6 [Source:HGNC Symbol;Acc:HGNC:6018] | 12460 | -0.730 | -0.0378 | Yes |
| 34 | NOTCH1 | notch receptor 1 [Source:HGNC Symbol;Acc:HGNC:7881] | 12555 | -0.776 | 0.0268 | Yes |
Table: GSEA details [plain text format]

  

Fig 2: KEGG\_PRION\_DISEASES      
 Blue-Pink O' Gram in the Space of the Analyzed GeneSet

  

Fig 3: KEGG\_PRION\_DISEASES: Random ES distribution      
 Gene set null distribution of ES for **KEGG\_PRION\_DISEASES**
